# Carbamazepine Enhances Adipogenesis by Inhibiting Wnt/β-Catenin Expression

**DOI:** 10.3390/cells8111460

**Published:** 2019-11-18

**Authors:** Dong Uk Im, Sang Chon Kim, Gia Cac Chau, Sung Hee Um

**Affiliations:** 1 Department of Molecular Cell Biology, Samsung Biomedical Research Institute, Sungkyunkwan University School of Medicine, Suwon, Gyeonggi-do 16419, Korea; ldw4545@gmail.com (D.U.I.); chaugiacac@gmail.com (G.C.C.); 2 Department of Health Sciences and Technology, Samsung Advanced Institute for Health Sciences and Technology, Samsung Medical Center, Sungkyunkwan University, Seoul 06351, Korea; karls22@nate.com; 3 Biomedical Institute Convergence at Sungkyunkwan University, Suwon, Gyeonggi-do 16419, Korea

**Keywords:** carbamazepine, obesity, adipocyte differentiation, adipogenesis, Wnt/β-catenin

## Abstract

Carbamazepine is a drug that is widely used in the treatment of epilepsy and bipolar disorder. The prevalence of obesity in patients treated with carbamazepine has been frequently reported. However, whether carbamazepine affects adipogenesis, one of the critical steps in the development of obesity, remains unclear. Here, we show that carbamazepine increased the expression levels of peroxisome proliferator-activated receptor γ (PPARγ), CCAAT/enhancer-binding protein β (C/EBPβ), and fatty acid synthase (FASN) in 3T3-L1 cells. Notably, carbamazepine inhibited the expression levels of β-catenin, a negative regulator of adipogenesis, leading to enhanced adipogenesis. Conversely, β-catenin overexpression abolished the effect of carbamazepine on adipogenic gene expression. However, depletion of β-catenin further enhanced PPARγ expression. In addition, carbamazepine reduced β-catenin expression by lowering the levels of phospho-low density lipoprotein receptor-related protein 6 (p-LRP6) and phospho-glycogen synthase kinase 3β (p-GSK3β) in Wnt/β-catenin signaling. Moreover, carbamazepine reduced Wnt mRNA expression and decreased the promoter activities of TCF, the target of β-catenin during adipogenesis. These results suggest that carbamazepine enhances adipogenesis by suppressing Wnt/β-catenin expression, indicating its potential effects on obesity-related metabolism.

## 1. Introduction

Carbamazepine, a dibenzazepine compound with tricyclic structure, has been used as a prophylactic agent for the treatment of bipolar disorder [1]. Carbamazepine preferentially binds to the voltage-gated sodium channel in neurons, blocks the sodium channel and stabilizes neuronal signaling by interfering with the secretion of neurotransmitters such as gamma-aminobutyric acid type A (GABAA) and glutamate [2,3,4]. In children with seizure disorder, the rate of overweight/obese subjects is significantly increased from 23% to 38.5% after carbamazepine treatment for 8 months at a concentration of 800 mg/day [5]. Carbamazepine treatment of 600–1000 mg/day for 8 weeks increases body weight from 14.6% to 22.4% in epileptic patients [6]. When administered to primary idiopathic epileptic children, carbamazepine treatment with 400–800 mg/dL for 2–10 years increases body mass index by 15% [7]. Levels of lipoprotein(a) (Lp(a)) (total and LDL cholesterol, triglycerides) in 20 healthy male volunteers treated with 800 mg of carbamazepine daily for 69 days are significantly increased from 14 to 19.8 mg/dL [8]. Although carbamazepine promotes weight gain in patients, the pathophysiological cause of this adverse effect remains unclear [5,9]. 

Recent studies have revealed that treatment with carbamazepine increases the levels of total cholesterol (TC), high-density lipoprotein (HDL) cholesterol, low-density lipoprotein (LDL) cholesterol, and triglyceride concentrations in serum without significantly affecting circulating insulin or leptin levels, suggesting the effect of carbamazepine in cholesterol metabolism [7,10,11]. Adipose tissue forms the major cholesterol pool of the human body [12]. Moreover, Wnt/β-catenin signaling cascade activation is persuaded by cholesterol degradation [13]. However, the relevance of carbamazepine treatment and the incidence of obesity linked to deregulated lipid homeostasis have not yet been fully understood.

Obesity represents an imbalance between energy intake and consumption, leading to increased sizes and numbers of adipose tissues. It is linked to the development of diabetes and cardiovascular diseases [14,15,16]. Pre-adipocytes are capable of propagating and differentiating into mature adipocytes that will determine the number of fat cells throughout their entire lifespan [14,15], which suggests that pre-adipocyte differentiation is one of the critical steps in the development of obesity [17]. The size of the fat cell depends on the level of lipid accumulation in adipocytes [14]. The mass of adipose tissue can be increased by adipocyte differentiation from pre-adipocytes to mature adipocytes or by lipid accumulation [18].

Adipogenesis is a cellular differentiation process that includes the formation of pre-adipocytes from mesenchymal stem cells and their differentiation into adipocytes [19]. The molecular mechanism of adipocyte differentiation has been widely studied using pre-adipocyte culture systems, including 3T3-L1 cell line [20]. In response to hormonal stimulation of adipogenesis, expression levels of transcription factors such as CCAAT/enhancer-binding protein β (C/EBPβ) and C/EBPδ are rapidly and temporarily induced [19,21]. These transcription factors enhance the expression of key adipogenic transcription factors C/EBPα and peroxisome proliferator-activated receptor γ (PPARγ) that can synergistically induce the expression of one another [21]. Furthermore, various factors such as sterol regulatory element-binding protein 1 (SREBP1), Krupel-like factors (KLFs), and Wingless-type MMTV integration site family members (Wnts) affect the differentiation of pre-adipocytes to mature adipocytes [19,21]. However, whether carbamazepine has any effect on the expression of these factors or adipocyte differentiation is not fully understood yet. 

In this study, we found that treatment with carbamazepine promoted the differentiation of 3T3-L1 pre-adipocytes and inhibited the expression levels of Wnt/β-catenin, a negative regulator of adipogenesis, suggesting that carbamazepine might have novel pro-adipogenic roles in the development of obesity. 

## 2. Materials and Methods

### 2.1. Cell Culture and Adipocyte Differentiation

The 3T3-L1 cells were maintained in Dulbecco’s modified Eagle’s medium (DMEM) with 10% calf serum, seeded onto 6-well-plates and cultured for 2 days. After that, pre-adipocytes were maintained in DMEM with 10% FBS. For adipocyte differentiation, pre-adipocytes were treated with DMI (dexamethasone, 3-isobutyl-1-methylxanthine, insulin) cocktail containing 1 μM dexamethasone (DEX), 0.5 mM 3-isobutyl-1-methylxanthine (IBMX), and 5.0 μg/mL insulin for 2 days. Cells were further incubated with 5.0 μg/mL insulin and treated with fresh media every 2 days. The differentiation process to mature adipocyte was completed after 6 days of differentiation. Human embryonic kidney (HEK) 293T cells were maintained in DMEM supplemented with 10% FBS in 5% CO_2_ at 37 °C, and used for luciferase assay.

### 2.2. Drug Treatment

The carbamazepine was purchased from Sigma-Aldrich (St. Louis, MO, USA). The stock concentration of carbamazepine was 100 mM, dissolved in 100% ethanol. The stock solution was diluted to a concentration of 80 μM with culture medium, and serially diluted to concentration of 40, 20, and 10 μM before use. We used an ethanol solution for vehicle (Veh) treatment, and carried out serial dilution in the same way as carbamazepine solution. Ethanol levels were kept below 0.5% in all cells, which did not affect differentiation. 3T3-L1 cells were treated with carbamazepine on day 0 of differentiation process. When the differentiation medium was replaced at 2-day intervals, carbamazepine was added together at a similar concentration. 

### 2.3. Oil Red O Staining

The Oil Red O powder was purchased from Sigma-Aldrich (St. Louis, MO, USA). Fully differentiated cells were fixed with 10% formalin for 1 h at room temperature (RT), rinsed with 60% isopropanol, and stained with filtered Oil Red O for 20 min. These cells were then visualized under a light microscope. The absorbance of the Oil Red O eluted by adding 100% isopropanol was measured at 500 nm wavelength by spectrophotometry.

### 2.4. Immunoblotting

Whole cell extracts were fractionated by SDS-PAGE, and transferred to a polyvinylidene fluoride (PVDF) membrane using a transfer apparatus, according to the manufacturer’s protocol (Bio-Rad, Hercules, CA, USA). The membranes were incubated with primary antibodies in Tris-Buffered Saline, and 0.1% Tween 20 detergent (TBS-T) containing 3% bovine serum albumin for overnight at 4 °C. After washing three times for 10 min with TBS-T, the membranes were incubated with secondary antibodies for 1 h at RT, followed by washing thrice with TBS-T, and developed with ECL system (Thermo, Waltham, MA, USA), according to the manufacture’s protocol. 

Antibodies against active β-catenin (cat. 05-665) from Merck Millipore, β-catenin (cat. 610154) and FASN (cat. 610963) from BD Bioscience, IRS1 (cat. 06-248) from Upstate Biotechnology (Billerica, MA, USA), Glut4 (cat. ab652) from Abcam (Boston, MA, USA), Flag (cat. F1804) from Sigma (St. Louis, MO, USA), p-AKT (cat. 9271), AKT (cat. 9272), p-LRP6 (cat. 2567), AXIN (cat. 2087), p-GSK3β (cat. 9336), GSK3β (cat. 12456), and IR (cat. 3025) from Cell Signaling Technology (Danvers, MA, USA). C/EBPβ (cat. sc-7962), PPARγ (cat. sc-7273), α-tubulin (cat. sc-5286), anti-rabbit IgG HRP (cat. sc-2030), anti-mouse IgG HRP (cat. sc-2005), and anti-goat IgG HRP (cat. sc-2020) from Santa Cruz Biotechnology (Paso Robles, CA, USA) were used. Signals were detected using chemiluminescent substrates (Thermo, Chelmsford, MA, USA).

### 2.5. Cell Viability Assay

The 3T3-L1 cells were seeded onto 96-well-plates and maintained in growth media for 24 h. The medium was replaced and cells were incubated with various concentrations of carbamazepine for 24 h. Cells were further incubated at 37 °C for 1 h with 1 mg/mL 3-(4,5-dimethylthiazol-2-yl)-2,5-diphenyltetrazolium bromide (MTT). Precipitates were dissolved with 200 μL DMSO. Cell viability was then measured at 570 nm wavelength using a microplate reader.

### 2.6. RNA Isolation, Reverse Transcriptase PCR (RT-PCR)

Total RNA was extracted using Trizol reagent (Invitrogen, Waltham, MA, USA), according to the manufacturer’s instructions. The cDNA synthesis was carried out with 1 μg of total RNA using the RevertAid First cDNA Synthesis kit (Thermo). Gene expression was determined by RT-PCR (SimpliAmp Thermal Cycler, Applied Biosystem, Foster City, CA, USA) using gene specific primers. Table 1 lists the primer sequences used for RT-PCR.

### 2.7. Quantitative Real-Time PCR (qRT-PCR)

The quantitative PCR analysis was performed with QuantStudio 6 Flex System Applied Biosystem (Carlsbad, CA, USA). Table 2 lists the primer sequences used for qRT-PCR. The PCR mixture contains cDNA (2 μg), SYBR green qPCR PreMIX (Enzynomics, Seoul, Korea) primers, and ultrapure RNase free water. PCR reactions were subjected to 45 cycles of 95 °C for 15 min, 95 °C for 15 s, and 60 °C for 1 min. The relative mRNA expression levels were normalized to glyceraldehyde-3-phosphate dehydrogenase (GAPDH), which was used as an internal control.

### 2.8. RNA Interference Experiment and Transfection

The β-catenin siRNA (si-β-catenin) and non-silencing siRNA (si-NS) were purchased from Macrogen (Seoul, Korea). For siRNA transfection, pre-adipocytes were seeded onto 6-well-plate for 24 h. Cells were transfected with either si-β-catenin or si-NS at a concentration of 50 nM using Lipofectamine RNAi max (Invitrogen, Waltham, MA, USA), according to the manufacturer’s protocol. Table 3 lists the siRNA sequences for β-catenin and non-silencing.

### 2.9. Transfection of β-catenin

The plasmid expressing β-catenin was purchased from Addgene (Watertown, MA, USA). For gene overexpression, cells were transfected with plasmids expressing either β-catenin or pcDNA3 (vector) using Lipofectamine 2000 (Invitrogen), according to the manufacturer’s protocol.

### 2.10. Luciferase Assay

The HEK293T cells were transfected with reporter vector and expression plasmid using TransIT-LT1 (Mirus, Singapore). Cells were lysed with cell lysis buffer (Promega, Madison, WI, USA) at two days after addition of differentiation stimulus. Luciferase activity was determined by luminometer (Promega) according to the manufacturer’s instruction.

### 2.11. Data and Statistical Analysis

Data are presented as mean ± standard error of the mean (SEM). The main and interactive effects were analyzed using Student’s *t*-test (unpaired, two-tail). Differences between individual group means were analyzed using two-tailed *t*-tests. *P* value of less than 0.05 was considered significant.

## 3. Results

### 3.1. Carbamazepine Enhances Adipocyte Differentiation in 3T3-L1 Cells

**Figure 1:**
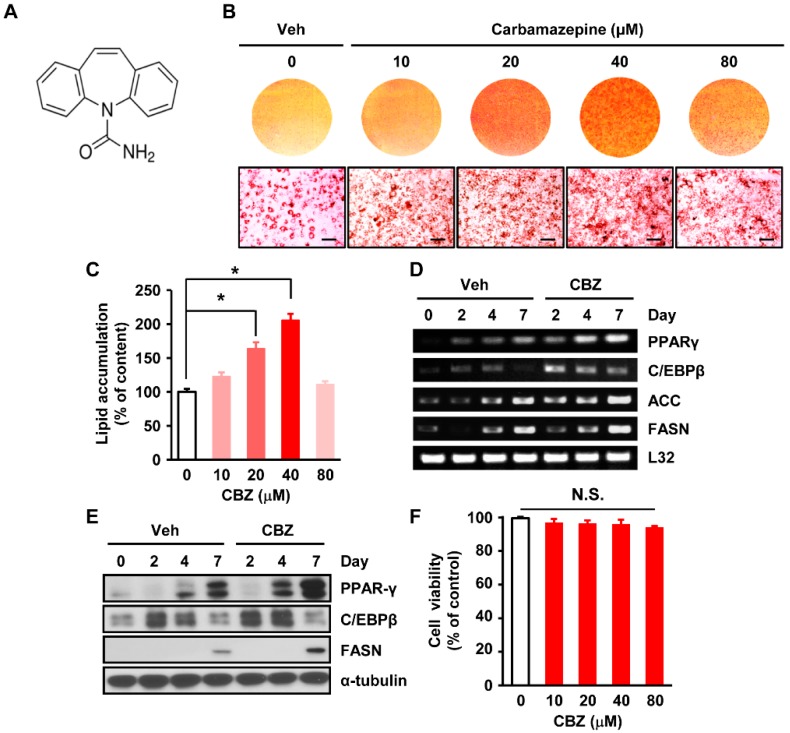
Carbamazepine increases adipocyte differentiation in 3T3-L1 cells. (**A**) Chemical structure of carbamazepine (CBZ) [22]. (**B**) 3T3-L1 cells were treated with vehicle or carbamazepine at the indicated concentration. At day 7, differentiated cells were stained with Oil Red O. (Top) Photomicrographs of entire wells; (Bottom) Photomicrographs of stained cells (scale bar 100 μm). (**C**) Lipid accumulation in cells treated with different concentrations of carbamazepine. Absorbance of wells was measured by spectrophotometry and normalized with DNA content (*n* = 5 per group). (**D**) Cells were treated with vehicle or 40 μM carbamazepine. RNA samples at the indicated time points were prepared for RT-PCR. (**E**) Cells were differentiated until the indicated time point and levels of protein expression were analyzed by western blot. (**F**) Viability of 3T3-L1 cells treated carbamazepine for 24 h was analyzed by MTT assay (*n* = 5 per group). N.S. = not significant. Values are mean ± SEM. * *P* < 0.05.

The structure of carbamazepine has previously been described (Figure 1A) [22]. To investigate the effect of carbamazepine on adipogenesis, 3T3-L1 cells were treated with vehicle or carbamazepine at various concentrations during adipocyte differentiation for a week. Carbamazepine and control vehicle were dissolved in 100% ethanol, and serially diluted in culture medium. Oil Red O staining revealed that the number and size of lipid droplets were markedly increased in carbamazepine treated cells, compared to those in vehicle-treated cells (Figure 1B). The enhancing effect of carbamazepine on adipogenesis was first observed at concentration of 10 μM. The highest level of lipid accumulation was observed after treatment with carbamazepine at 40 μM. However, a high concentration of carbamazepine (80 μM) did not further enhance lipid accumulation (Figure 1C). These results suggest that carbamazepine enhances adipogenesis in 3T3-L1 cells.

### 3.2. Carbamazepine Increases Expression Levels of Genes Related to Adipogenic Transcription Factors and Lipogenic Enzymes

We next investigated how carbamazepine affected adipocyte differentiation in 3T3-L1 cells. Adipogenic transcription factors such as adipocyte protein 2 (aP2), C/EBPα, C/EBPβ, PPARγ, and lipogenic enzymes, including acetyl-CoA carboxylase (ACC) and FASN, are critical for adipocyte differentiation [19,21]. Thus, we examined mRNA and protein expression levels of these factors after carbamazepine treatment for 2, 4, or 7 days. The mRNA expression levels of *PPARγ*, *C/EBPβ*, *ACC*, and *FASN* were increased by treatment with carbamazepine in a time-dependent manner (Figure 1D). Protein levels of PPARγ were also increased by carbamazepine in a time-dependent manner. Protein levels of FASN were enhanced in the late stage (day 7), whereas those of C/EBPβ were increased in the early stage (days 2–4) of adipocyte differentiation upon treatment with carbamazepine (Figure 1E and Figure S2A). After 3T3-L1 cells were treated with carbamazepine at different concentrations, cell viability was assessed by MTT assay. At concentration up to 80 μM, cell viability was not significantly affected by carbamazepine (Figure 1F). When the concentration of carbamazepine was above 100 μM, survival rates of cells were significantly reduced (Figure S1). Taken together, these results suggest that treatment with carbamazepine enhances lipid accumulation and adipocyte differentiation in 3T3-L1 cells by up-regulating the expression levels of adipogenic transcription factors and lipogenic enzymes. 

### 3.3. Carbamazepine Enhances Adipogenesis at Late Phase of the Differentiation Process

We next determined the critical time point of carbamazepine action. Confluent 3T3-L1 cells were exposed to DMI with or without carbamazepine for various time periods during adipogenesis. 

**Figure 2:**
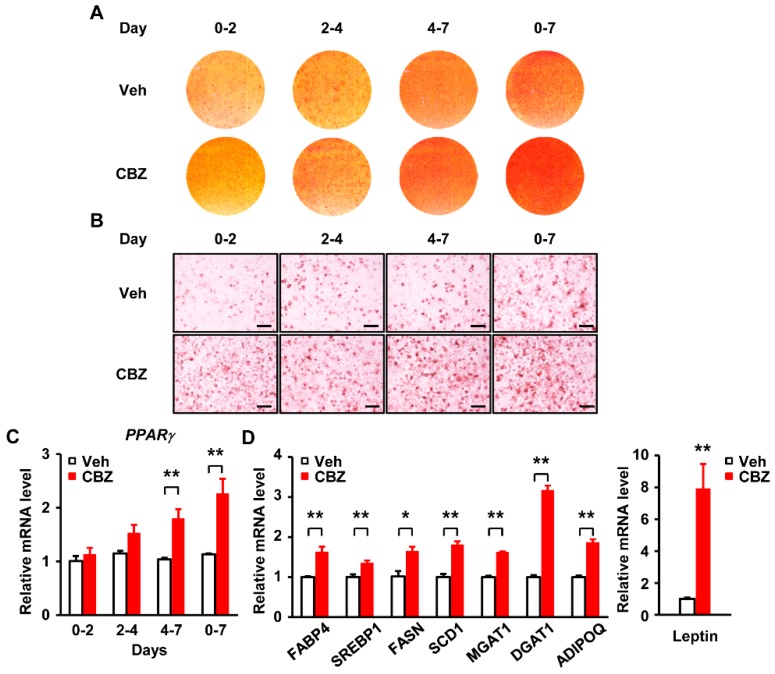
Carbamazepine enhances late stage of adipocyte differentiation. (**A**) and (**B**) 3T3–L1 cells were treated with vehicle or 40 μM carbamazepine for the indicated time. At day 7 after differentiation, cells were stained with Oil Red O. (**A**) Photomicrographs of entire wells. (**B**) Photomicrographs of Oil Red O stained cells (scale bar, 100 μm). (**C**) Peroxisome proliferator-activated receptor γ (*PPARγ*) mRNA levels after carbamazepine treatment were analyzed by qRT-PCR (*n* = 5 per group). (**D**) mRNA levels of genes involved in adipogenic transcription, lipid synthesis, and adipokine production after carbamazepine treatment were analyzed by qRT-PCR (*n* = 5 per group). Values are mean ± SEM. * *P* < 0.05; ** *P* < 0.01.

We divided the adipocyte differentiation process into four different phases: Early (days 0–2), intermediate (days 2–4), late (days 4–7), and throughout the 7-day period (complete) phase (Figure 2A,B). After carbamazepine treatment in late phase, mRNA levels of *PPARγ* were markedly increased (Figure 2C). Carbamazepine was more effective at late phase rather than at early phase (Figure 2C). These results suggest that carbamazepine primarily has effect on the late stage of adipocyte differentiation and enhances adipogenesis by controlling *PPARγ* expression. 

We further analyzed adipogenic gene expression in carbamazepine treated adipocytes using qPCR. We examined changes of gene expression levels in differentiated adipocytes treated with vehicle or carbamazepine for 7 days. mRNA levels of adipogenic transcription factors (such as *SREBP1*), lipid synthesis-related genes (such as *FASN*, stearoyl-Coenzyme A desaturase 1 (*SCD1*), acyl-CoA:monoacylglycerol acyltransferase (*MGAT1*), and acyl-CoA:diacylglycerol acyltransferase (*DGAT1*)), and adipokine related genes (such as adiponectin (*ADIPOQ*), fatty acid binding protein 4 (*FABP4*), and *Leptin*) in carbamazepine treated adipocytes were increased, compared to those in vehicle-treated cells (Figure 2D), suggesting that carbamazepine induces adipocyte differentiation by controlling the expression levels of adipogenic transcription factors and genes related to lipid synthesis. In addition, our results showed that the carbamazepine treatment promoted adipocyte differentiation, and increased the levels of insulin signaling proteins, such as Akt, insulin receptor (IR), insulin receptor substrate 1 (IRS1), and glucose transporter type 4 (Glut4) during differentiation (Figure S3A). However, carbamazepine treatment did not increase the expression levels of Akt phosphorylation in differentiated adipocytes (Figure S3B). These results suggest that carbamazepine increases adipogenesis with long-term insulin treatment, whereas it does not affect insulin sensitivity in fully differentiated adipocytes.

### 3.4. Carbamazepine Decreases β-catenin Signaling and Leads to Enhanced Adipogenesis

**Figure 3:**
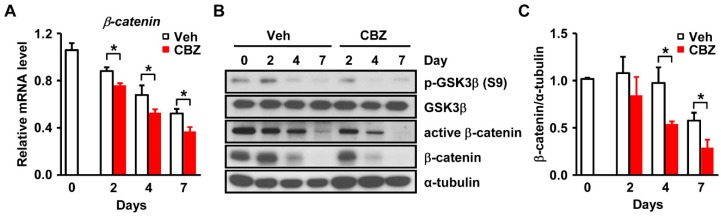
Carbamazepine decreases Wnt/β-catenin signaling. (**A**) mRNA levels of *β-catenin* after carbamazepine treatment at the indicated time points were determined by qRT-PCR (*n* = 5 per group). (**B**) Protein levels of Wnt/β-catenin signaling were evaluated by Western blot. (**C**) Densitometry represents relative protein levels of β-catenin (*n* = 5 per group). Values are mean ± SEM.* *P* < 0.05.

Among the factors involved in adipocyte differentiation, Wnt/β-catenin signaling is known to suppress adipogenesis by inhibiting the expression of PPARγ [23]. Conversely, inhibition of Wnt/β-catenin signaling induces pre-adipocyte differentiation [24,25,26]. We next examined whether β-catenin is involved in carbamazepine-induced *PPARγ* expression. Carbamazepine significantly decreased the mRNA levels of *β-catenin* in 3T3-L1 cells during adipocyte differentiation (Figure 3A). In carbamazepine treated 3T3-L1 cells, protein levels of β-catenin and de-phosphorylated β-catenin (the active form) were reduced compared to those in vehicle-treated cells (Figure 3B and Figure S2B). Phosphorylated β-catenin is the inactive form of β-catenin [27]. It is degraded in the cytoplasm [27]. De-phosphorylation of β-catenin increases its stability in the cytoplasm [28]. β-catenin can enter the nucleus and bind to the T-cell factor (TCF)/Lymphoid enhancer-binding factor (LEF) promoter to increase its activity [27,28]. 

We then investigated Wnt/β-catenin signaling in carbamazepine treated cells. Wnt/β-catenin signaling is initialized by binding of Wnt ligands to frizzled receptors and low-density lipoprotein receptor-related protein 6 (LRP6) co-receptors [29]. Levels of phospho-glycogen synthase kinase 3β (p-GSK3β), β-catenin and active β-catenin were reduced in carbamazepine treated cells (Figure 3B). 

Lrp5/6 and Fizzled (FZD) act as receptors for Wnt signaling [30]. FZD binds to glycogen synthase kinase 3β (GSK3β) to interfere with the formation of GSK3β-AXIN complex, which induces the translocation of β-catenin into the nucleus to inhibit adipocyte differentiation [30]. Our results further revealed that when cells were treated with carbamazepine, the level of p-GSK3β was decreased, resulting in reduced expression of β-catenin (Figure 3B,C). Thus, treatment with carbamazepine reduced the levels of p-GSK3β, which eventually decreased the expression of β-catenin and inhibited Wnt/β-catenin signaling, thereby promoting adipogenesis. 

**Figure 4:**
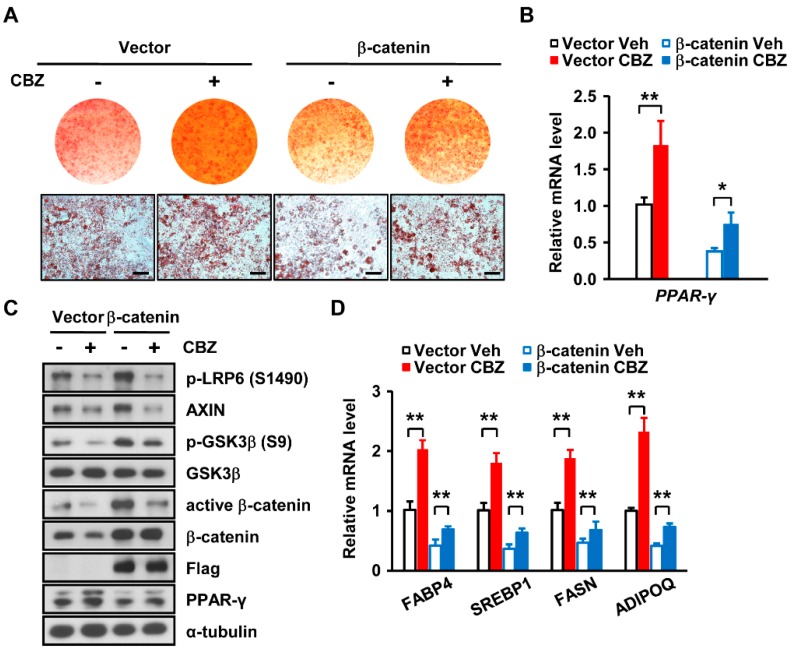
β-catenin overexpression abolishes the effect of carbamazepine on adipogenesis. (**A**) Cells treated with vehicle or 40 μM carbamazepine were differentiated after β-catenin overexpression and stained with Oil Red O. (Top) Photomicrographs of entire wells; (Bottom) Photomicrographs of stained cells (scale bar, 100 μm). (**B**) *PPARγ* mRNA levels were analyzed by qRT-PCR (*n* = 5 per group). (**C**) Protein levels of Wnt/β-catenin signaling and PPARγ in 3T3-L1 cells overexpressing β-catenin after carbamazepine treatment were analyzed by Western blot. (**D**) mRNA levels of adipogenic genes in 3T3-L1 cells overexpressing β-catenin after carbamazepine treatment were analyzed by qRT-PCR (*n* = 5 per group). Values are mean ± SEM. * *P* < 0.05; ** *P* < 0.01.

To further investigate the effect of carbamazepine on β-catenin expression during adipocyte differentiation, we assessed adipogenesis after overexpressing *β-catenin* in 3T3-L1 cells. Overexpression of *β-catenin* abolished the positive effect of carbamazepine on adipocyte differentiation (Figure 4A), leading to a decrease in *PPARγ* expression (Figure 4B,C). Expression levels of p-LRP6, p-GSK3β, β-catenin and active β-catenin were also reduced in cells overexpressing β-catenin after carbamazepine treatment (Figure 4C and Figure S2C). In addition, carbamazepine increased expression levels of adipogenic transcription factors, genes related to lipid synthesis, and adipokine, although during β-catenin overexpression, these levels were decreased (Figure 4D). This result suggests that carbamazepine-induced suppression of β-catenin expression increases PPARγ expression and leads to enhanced adipocyte differentiation. 

### 3.5. Depletion of β-catenin Further Enhances Carbamazepine-Induced Adipogenesis

**Figure 5:**
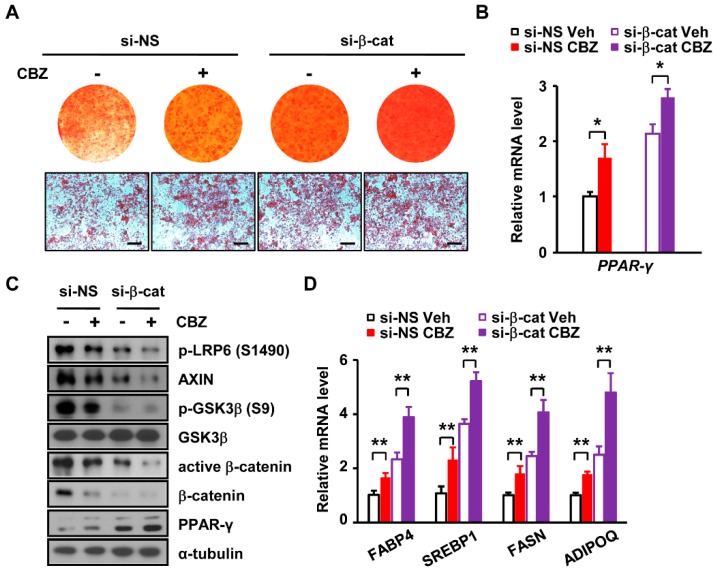
Depletion of β-catenin further enhances carbamazepine-induced adipogenesis. (**A**) Cells treated with vehicle or carbamazepine were differentiated after β-catenin knockdown and then stained with Oil Red O. (Top) Photomicrographs of entire wells; (Bottom) Photomicrographs of stained cells (scale bar, 100 μm). (**B**) qRT-PCR analysis of *PPARγ* mRNA levels (*n* = 5 per group). (**C**) Protein levels of Wnt/β-catenin signaling and PPARγ in β-catenin depleted 3T3-L1 cells after carbamazepine treatment were analyzed by Western blot. (**D**) mRNA levels of adipogenic genes in β-catenin depleted 3T3-L1 cells after carbamazepine treatment were analyzed by qRT-PCR (*n* = 5 per group). Values are mean ± SEM. * *P* < 0.05; ** *P* < 0.01.

We next examined whether the si-RNA mediated depletion of β-catenin affects carbamazepine-induced adipogenesis. Non-silencing si-RNA (si-NS) was used as a control for the si-RNA against β-catenin (si-β-catenin). si-NS and si-β-catenin transfected cells were treated with vehicle or carbamazepine. The positive effect of carbamazepine on adipocyte differentiation was further increased in β-catenin knockdown cells (Figure 5A). After depletion of β-catenin, the number and size of lipid droplets in carbamazepine treated cells were increased, compared to those in non-silencing treated cells (Figure 5A), suggesting that carbamazepine induces adipogenesis by decreasing β-catenin expression. In addition, mRNA expression levels of *PPARγ* were further increased in β-catenin depleted cells upon the treatment with carbamazepine (Figure 5B). The protein expression levels of β-catenin were reduced about 80% by si-β-catenin (Figure 5C and Figure S2D). Depletion of β-catenin resulted in higher PPARγ protein levels in carbamazepine treated cells (Figure 5C and Figure S2D). Decreased levels of p-GSK3β, β-catenin and active β-catenin were further aggravated by β-catenin depletion (Figure 5C). In addition, carbamazepine-induced mRNA levels of adipogenic genes were further increased by lowering levels of β-catenin (Figure 5D).

### 3.6. Carbamazepine Inhibits the Activity of Wnt/β-catenin During Adipocyte Differentiation

**Figure 6:**
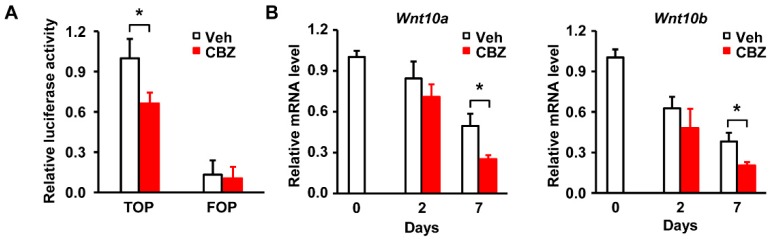
Carbamazepine inhibits the activity of β-catenin and Wnt expression in 3T3-L1 cells. (**A**) Cells were transfected with TCF reporter plasmid (TOP) or TCF binding site mutant plasmid (FOP) and then treated with carbamazepine. Luciferase activities of β-catenin were measured using a luminometer (*n* = 5 per group). (**B**) Relative mRNA levels of *Wnt 10a* and *Wnt 10b* in carbamazepine treated cells. mRNA levels of were expressed as the relative ratio to that of day 0 (*n* = 5 per group). Values are mean ± SEM. * *P* < 0.05.

We next examined whether carbamazepine affects the transcriptional activity of β-catenin. Considering that β-catenin is associated with DNA-bound TCF to serve as its co-activator for regulating Wnt-responsive genes [27] and that TCF inhibits adipocyte differentiation by reducing the expression levels of *C/EBPα* and *PPARγ* [31], we measured the binding of β-catenin to TCF in carbamazepine treated cells, using luciferase reporter assay. TOP (TCF receptor plasmid) /FOP (TCF binding site mutant plasmid) flash assay revealed that the binding of β-catenin to TCF was reduced upon carbamazepine treatment, suggesting that carbamazepine inhibits the transcriptional activity of β-catenin (Figure 6A). FOP is a negative control that does not bind to TCF due to its mutation. Our results showed no change of FOP flash after carbamazepine treatment (Figure 6A). 

Wnt is an upstream regulator of β-catenin [27,32], that increases the stability of β-catenin to progress signaling in 3T3-L1 cells [27,33,34]. Among Wnt families, Wnt10 decreases the expression of adipogenesis-related genes, and is closely associated with adipocyte differentiation [35]. 

Wnt increases the expression of β-catenin and leads to its association with TCF, thereby decreasing the expression of *PPARγ* and eventually resulting in the inhibition of adipogenesis [24]. Considering the role of Wnt in controlling β-catenin during adipocyte differentiation, we examined whether carbamazepine affects Wnt expression. Treatment with carbamazepine resulted in significant decrease of *Wnt10a* and *Wnt10b* mRNA expression levels at day 7, compared with those at day 2 after the initiation of differentiation (Figure 6B), suggesting an inhibitory effect of carbamazepine on Wnt expression at late stage of adipogenesis. Taken together, these results indicate that carbamazepine enhances adipocyte differentiation by inhibiting Wnt/β-catenin expression in 3T3-L1 cells. 

## 4. Discussion

Carbamazepine is commonly used for treating seizure disorder, epilepsy, neuropathic pain, and bipolar disorder as a mood stabilizer [36]. Several studies have reported the occurrence of obesity and metabolic disorder in patients administered with carbamazepine [5,11,37,38,39]. Obesity and mood disorders share many features, that include phenomenological symptoms such as excess eating, physical inactivity, and subsequent weight gain [40]. The process of fat gain involves increased adipogenesis [21]. However, it is not yet well understood how carbamazepine is related to obesity. Here, we found that carbamazepine enhanced adipocyte differentiation. 

Adipogenesis is a critical process for controlling lipid homeostasis, energy balance, and obesity [15]. Adipocyte differentiation is regulated by a highly organized cascade involving numerous transcription factors such as PPARγ, C/EBPα, and C/EBPβ [19,21] that control the expression of lipogenic genes such as *ACC* and *FASN* [41]. In this study, we showed that carbamazepine increased mRNA expression levels of *PPARγ* and *C/EBPβ* and lipogenic enzymes such as *ACC* and *FASN* in 3T3-L1 cells, indicating that carbamazepine enhances lipid accumulation by controlling the expression levels of adipogenic transcription factors and lipogenic enzymes. 

Adipocyte differentiation is divided into three stages: Early, middle, and late stages [20]. Specific transcription factors are involved in each stage [21]. Our analysis showed that treatment with carbamazepine at 40 μM enhanced adipogenesis and lipid accumulation at the late stage of differentiation through PPARγ. Moreover, the expression levels of adipogenic genes (such as *SREBP*)*,* genes related to lipid synthesis (such as *FASN*, *SCD1*, *MGAT1*, and *DGAT1*)*,* and adipokine genes (including *Adipoq*, *leptin*, and *FABP4*) were increased after carbamazepine treatment. In contrast, Turpin et al. [42] have reported that carbamazepine treatment at a concentration of 500 μM inhibits adipocyte differentiation and suppresses the expression levels of adipogenic transcription factors (such as *PPARγ*, *C/EBPα*, and *C/EBPβ*) and lipogenic enzymes (such as *ACC*, and *FASN*) in 3T3-L1, 3T3-F442A, and T37i cells. However, the concentration of carbamazepine used in their study was very high (500 μM). The high dose of carbamazepine (500 μM) might affect cell viability. Our analysis revealed that 40 μM of carbamazepine did not affect cell viability. However, carbamazepine at a concentration greater than 100 μM significantly reduced cell viability. These results suggest that depending on its dosage, carbamazepine has a distinct effect on adipocyte differentiation and cell viability.

Expression of insulin signaling is known to increase as adipocyte differentiation progresses [43]. Our results also indicated that on day 7 of differentiation, carbamazepine treatment increased the expression of insulin signaling. However, after carbamazepine treatment for 30 min, there were no significant differences in insulin sensitivity in fully differentiated adipocytes. Presumably, carbamazepine might have promoted adipocyte differentiation by reducing β-catenin expression through Wnt/β-catenin signaling, without affecting insulin signaling. Consistent with this, other studies have demonstrated that β-catenin and Wnt10b do not affect insulin-induced Akt phosphorylation and IRS1 expression [26,44,45]. In addition, Jaideep et al. have shown that there are no significant changes at the phosphorylation levels of Akt after insulin treatment between hepatocytes from β-catenin knockout and transgenic mice [45], suggesting that the expression levels of β-catenin do not absolutely correlate with the levels of insulin-mediated Akt phosphorylation. These results indicate that the expression of insulin signaling was increased due to enhanced differentiation, along with the increase in the number of adipocytes by carbamazepine treatment. Thus, carbamazepine increases adipogenesis through Wnt/β-catenin expression, rather than affecting insulin sensitivity in differentiated adipocytes. 

The mechanism by which carbamazepine enhances adipogenesis is mostly unknown. Our results showed that carbamazepine treatment decreased β-catenin expression levels in 3T3-L1 cells. Among pathways that regulate the expression of β-catenin, the canonical pathway is well known [27]. This pathway is regulates the expression of β-catenin via cascade signaling and controlled by the binding of Wnt ligand [32,46]. Wnt increases the activity of β-catenin by enhancing GSK3β, AXIN, adenomatous polyposis coil gene product (APC), and casein kinase 1 (CK1); these are known as AXIN complex in combination [27,28]. Our results revealed that carbamazepine enhanced adipocyte differentiation by downregulating expression levels of β-catenin, a negative regulator of adipogenesis. Moreover, we found that overexpression of β-catenin abolished the effect of carbamazepine on adipocyte differentiation and adipogenic gene expression, whereas β-catenin depletion further enhanced adipogenesis. These results suggest that carbamazepine treatment may reduce the expression of β-catenin through the Wnt/β-catenin pathway and eventually increase the expression of adipogenic genes to promote adipocyte differentiation.

Recent studies have reported that Wnt ligand binds to LRP5/6 and FZD receptor to activate the Wnt/β-catenin signaling pathway [29]. Our results showed that after carbamazepine treatment, β-catenin signaling was down-regulated by reduced LRP6 activity. Considering that carbamazepine treatment reduced the expression levels of pGSK3β and pLRP6, down-regulating β-catenin signaling would promote adipocyte differentiation. Furthermore, our results revealed that carbamazepine treatment reduced the expression levels of AXIN. The Wnt-FZD-LRP6 complex induces LRP6 phosphorylation and recruitment of active AXIN complex to LRP6 [47]. These proceedings impede AXIN-mediated β-catenin degradation [48]. Thus β-catenin becomes stabilized [33], and after reaching the nucleus, activates Wnt target gene expression [27]. Others and our results suggest that carbamazepine treatment may promote adipogenesis by inhibiting Wnt/β-catenin signaling.

Our results showed that Wnt expression was decreased in mature adipocytes and further reduced by carbamazepine treatment. Suppression of Wnt expression by carbamazepine may involve various mechanisms. When pre-adipocytes differentiate into mature adipocytes, carbamazepine treatment might have further promoted adipogenesis and increased lipid accumulation of mature adipocytes. One hypothesis is that the expression of Wnt itself might be decreased due to elevated lipid accumulation in mature adipocytes [49]. Our results showed that carbamazepine treatment induced a quantitative increase in mature adipocyte, resulting in decreased mRNA expression of *Wnt*. It would be of interest in the future to determine whether carbamazepine suppresses Wnt expression via the inhibition of LRP6 phosphorylation.

Wnt factors are a group of extracellular signaling molecules. Among them, Wnt10b is expressed in dividing and confluent pre-adipocytes [26]. Its expression is decreased during differentiation [26]. In adipocytes, Wnt10b is an intracellular regulator that inhibits the expression of C/EBPα and PPARγ, resulting in the prevention of adipocyte formation [24,50]. Forced expression of Wnt10b in 3T3-L1 cells stabilizes free cytosolic β-catenin and inhibits adipogenesis [35]. Our results showed that carbamazepine decreased the mRNA expression levels of *Wnt10a*, *Wnt10b*, and *β-catenin* in 3T3-L1 cells. Consistent with our results, a study using mice overexpressing Wnt10b has revealed that Wnt10b inhibits adipocyte formation both in vitro and in vivo [26,27]. In the future, it needs to be determined whether targeting Wnt10 in obesity accompanied by carbamazepine treatment might be effective in a pre-clinical setting.

In conclusion, our results indicate that carbamazepine increases adipocyte differentiation by inhibiting Wnt/β-catenin expression. Based on our results, potential therapeutic strategies could be developed by targeting β-catenin signaling and its expression to control obesity in patients treated with carbamazepine. 

## Figures and Tables

**Table 1:** List of primers used RT-PCR.

Name	Forward (5′→3′)	Reverse (5′→3′)
*C/EBPβ*	GGCCAAGAAGACGGRGGACAA	TTCTTCTGCACGCGCTCGTTC
*PPARγ*	GGGTGAAACTCTGGGAGATTCTCC	CAGCAACCATTGGGTCAGCTCT
*FASN*	GCTTATTGATCAGTTATGTGGCC	CACGGAGTTGAGCCGCAT
*L32*	TGAAGCAGGCATCTGAGGG	CGAAGGTGGAAGAG TGGGAG

**Table 2:** List of primers used for real-time quantitative PCR (qRT-PCR).

Name	Forward (5′→3′)	Reverse (5′→3′)
*PPARγ*	TGT GGG GAT AAA GCA TCA GGC	CCG GCA GTT AAG ATC ACA CCT AT
*FABP4*	TGG AAG CTT GTC TCC AGT GA	AAT CCC CAT TTA CGC TGA TG
*SREBP1*	GGCACTGAAGCAAAGCTGAAT	GCAAGAAGCGGATGTAGTCGAT
*FASN*	ACCACTGCATTGACGGCCGG	GGGTCAGGCGGGAGACCGAT
*SCD1*	GGTGATGTTCCAGAGGAGGTACTAC	AGCGTGGGCAGGATGAAG
*MGAT1*	CTGGTTCTGTTTCCCGTTGT	TGGGTCAAGGCCATCTTAAC
*DGAT1*	GTGCACAAGTGGTGCATCAG	CAGTGGGATCTGAGCCATCA
*Adipoq*	GGAACTTGTGCAGGTTGGAT	GCTTCTCCAGGCTCTCCTTT
*Leptin*	CACACACGCAGTCGGTATCC	AGCCCAGGAATGAAGTCCAA
*Wnt10a*	CCACTCCGACCTGGTCTACTTTG	TGCTGCTCTTATTGCACAGGC
*Wnt10b*	ATCGCCGTTCACGAGTGTC	GGAAACCGCGCTTGAGGAT
*GAPDH*	GTCTTCCTGGGCAAGCAGTA	CTGGACAGAAACCCCACTTC

**Table 3:** List of siRNA sequences used for RNA interference.

Name	Sense (5′→3′)	Antisense (5′→3′)
*β-catenin*	UAAUGAAGGCGAACGGCAUUCUGGG	CCCAGAAUGCCGUUCGCCUUCAUUA
*Nonsilencing*	CCUCGUGCCGUUCCAUCAGGUAGUU	CUACCUGAUGGAACGGCACGAGGUU

## Supplementary Materials

The following are available online at https://www.mdpi.com/2073-4409/8/11/1460/s1, Figure S1: Effect of carbamazepine on viability of 3T3-L1 cells. Figure S2: Quantification of western blot data. Figure S3: Carbamazepine does not affect insulin-induced Akt phosphorylation in differentiated adipocytes.
