# Supplementary material for: Carbamazepine Enhances Adipogenesis by Inhibiting Wnt/β-Catenin Expression
Source: Cells. 2019 Nov 18;8(11):1460. doi: 10.3390/cells8111460 (PMC6912407; doi:10.3390/cells8111460)
Supplement: Supplementary file 1 [file cells-08-01460-s001.pdf]

Supplementary Figures

Carbamazepine Enhances Adipogenesis by Inhibiting Wnt/β-catenin Expression

Dong Uk Im[1,2], Sang Chon Kim[2], Gia Cac Chau[1], and Sung Hee Um[1,2,3,*]

[1] Department of Molecular Cell Biology, Samsung Biomedical Research Institute, Sungkyunkwan University School of Medicine, Suwon, Gyeonggi-do 16419, Korea; ldw4545@gmail.com (D.I.); chaugiacac@gmail.com (G.C.)
[2] Department of Health Sciences and Technology, Samsung Advanced Institute for Health Sciences and Technology, Samsung Medical Center, Sungkyunkwan University, Seoul 06351, Korea; karls22@nate.com (S.K.)
[3] Biomedical Institute Convergence at Sungkyunkwan University, Suwon, Gyeonggi-do, 16419, Korea
* Correspondence: shum@skku.edu; Tel: +82-31-299-6123, Fax: +82-31-299-6109

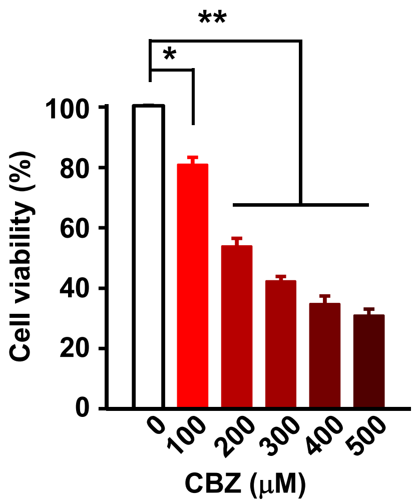

Figure S1. Effect of carbamazepine on the viability in 3T3-L1 cells.
Cell viability of 3T3-L1 cells treated with carbamazepine at various concentration for 24 h was analyzed by MTT assay (n = 3 per group). *t*-test, Values are mean ± SEM. *, $P < 0.05$; **, $P < 0.01$.

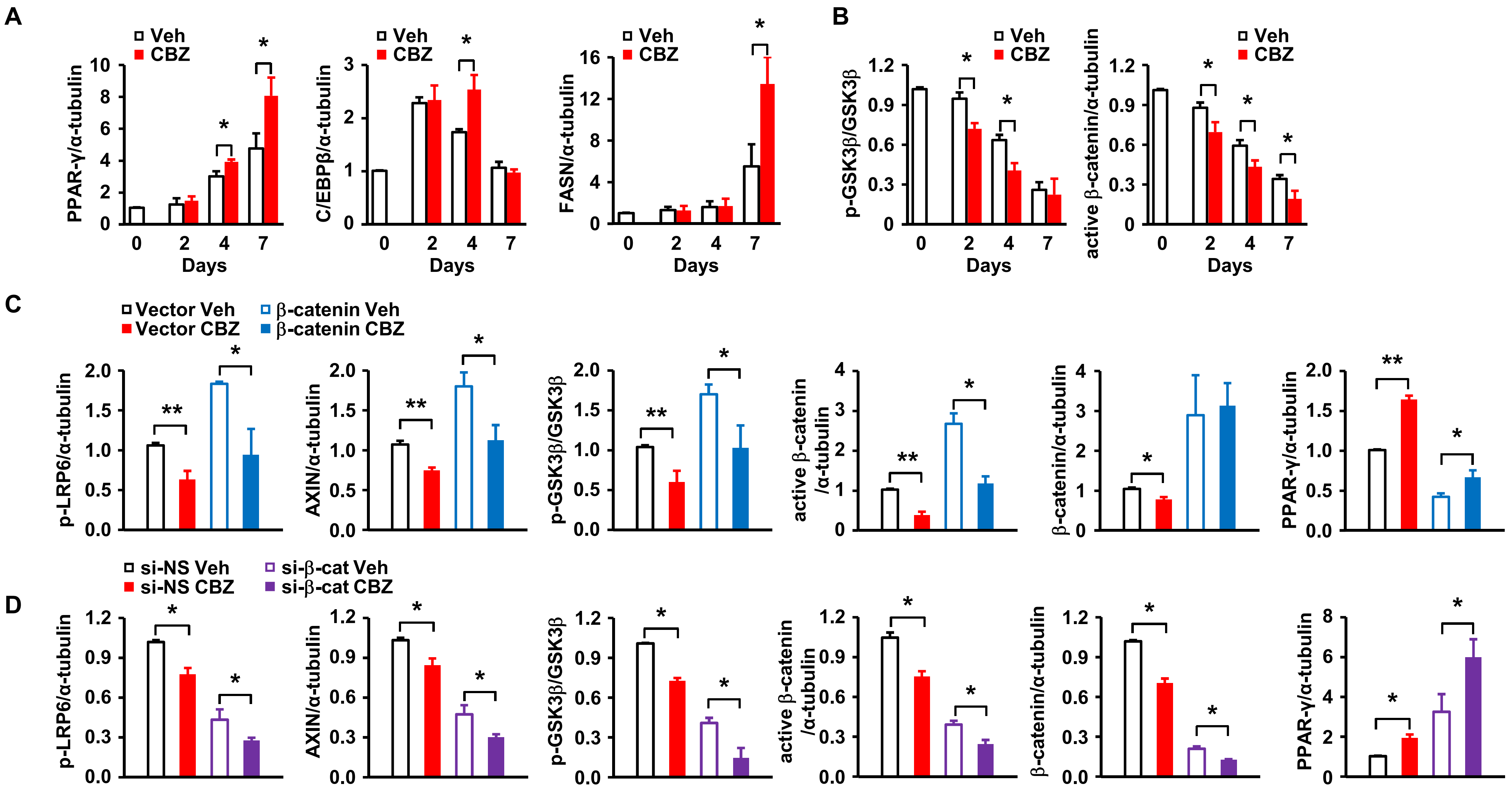

Figure S2. Quantification of western blot data.

Quantification of western blots from three independent experiments was performed using Image J software. Quantification analysis of (A) The relative levels of PPARγ/α-tubulin, C/EBPβ/α-tubulin and FASN/α-tubulin in Figure 1E (n=3 per group). (B) The relative levels of p-GSK3β/total GSK3β and active β-catenin/α-tubulin in Figure 3B (n=3 per group). (C) The relative levels of p-LRP6/α-tubulin, AXIN/α-tubulin, p-GSK3β/total GSK3β, active β-catenin/α-tubulin, β-catenin/α-tubulin and PPARγ/α-tubulin in Figure 4C (n=3 per group). (D) The relative levels of p-LRP6/α-tubulin, AXIN/α-tubulin, p-GSK3β/total GSK3β, active β-catenin/α-tubulin, β-catenin/α-tubulin and PPARγ/α-tubulin in Figure 5C (n=3 per group). *t*-test, Values are mean ± SEM. *, $P < 0.05$; **, $P < 0.01$.

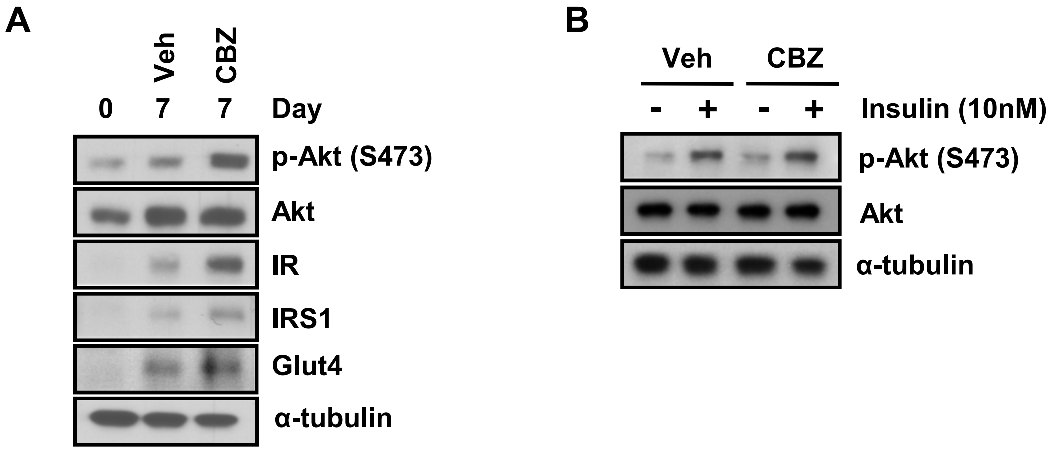

Figure S3. Carbamazepine does not affect insulin-induced Akt phosphorylation in differentiated adipocytes.
(A) Protein levels of insulin signaling after carbamazepine treatment during adipocyte differentiation. (B) Immunoblots showing insulin-induced phosphorylation of Akt in mature adipocytes after carbamazepine treatment for 30 min.
